# Biological roles and molecular mechanism of circular RNAs in epithelial-mesenchymal transition of gastrointestinal malignancies

**DOI:** 10.32604/or.2024.051589

**Published:** 2025-02-28

**Authors:** ZIYI FANG, YONGFU SHAO, MENG HU, JIANING YAN, GUOLIANG YE

**Affiliations:** 1 School of Basic Medical Sciences, Health Science Center, Ningbo University, Ningbo, 315211, China; 2 Department of Gastroenterology, The First Affiliated Hospital of Ningbo University, Ningbo, 315020, China

**Keywords:** Epithelial-mesenchymal transition, Circular RNAs (circRNAs), Gastrointestinal malignancies

## Abstract

Circular RNAs (circRNAs) are formed by splicing of precursor RNAs and covalently linked at the 5′ and 3′ ends. Dysregulated circRNAs are closely related to the epithelial-mesenchymal transition (EMT) of gastrointestinal malignancies. CircRNAs, including circRNA_0008717, circGOT1, circ-DOCK5, circVPS33B, circPVT1, circMET, circ-OXCT1, circ_67835, circRTN4, circ_0087502, circFNDC38, circ_PTEN1, circPGPEP1, and circ-E-Cad are involved in the EMT process of gastrointestinal malignancies through a variety of mechanisms, such as regulating EMT-inducing transcription factors, signaling pathways, and tumor microenvironments. Gastrointestinal (GI) malignancies are common malignant tumors worldwide, and the heterogeneity and easy metastasis of gastrointestinal malignancies limit the effectiveness of medical treatments. Therefore, investigating the molecular mechanisms involved in the pathogenesis of gastrointestinal malignancies is essential for clinical treatment. This article summarizes the biological roles and molecular mechanism of circRNAs in EMT of gastrointestinal malignancies, providing a theoretical basis for applying EMT-related circRNAs in targeted therapy.

## Introduction

Gastrointestinal (GI) malignancies are common malignant tumors found worldwide, that seriously endanger human health. According to 2020 global cancer statistics, gastrointestinal malignancies include gastric (GC), colorectal (CRC), esophageal (ESCC), hepatic (HCC), gallbladder, and pancreatic cancer. The incidence rate of these malignancies accounts for more than 50% of all cancers, and the mortality rate exceeds 35%, causing huge economic and social burdens worldwide [1]. Due to the insidious onset of gastrointestinal malignancies, nonspecific clinical manifestations, and rapid progression of the disease, most patients are already in the progressive stage at the time of diagnosis, and the best surgical window is missed. Additionally, the heterogeneity of gastrointestinal malignancies and their tendency to metastasize limit the effectiveness of neoadjuvant radiotherapy, molecular targeted therapy, immunotherapy, and other medical treatments for patients with clinically advanced tumors. Therefore, further understanding of the molecular mechanisms underlying the pathogenesis of gastrointestinal malignancies is of great significance for clinical treatment.

Epithelial-mesenchymal transition (EMT) is a reversible process in which polarized epithelial cells lose the attachment polarity of the basement membrane and the ability of intercellular tight and adhesion junctions in response to a few factors and are converted to mesenchymal cells with infiltrative and migratory abilities. EMT is strongly associated with tumor infiltration and migration and plays an important role in the metastasis of gastrointestinal malignancies [2]. Cancer cells with epithelioid morphology undergo remodeling and detach from the basement membrane with the programmed activation of EMT, transforming into mesenchymal cell morphology, with increased infiltration and migration capacity and spread of cancer cells to the periphery, promoting cancer metastasis [3]. During the EMT process, cadherin is an important molecular marker of EMT and plays an important biological effect. E-cadherin is a key protein mediating intercellular adhesion junctions, which inhibits metastasis by preventing β-cadherin from entering the nucleus of the cell and hindering the action of DNA-binding proteins [4]. When the activation of the EMT process is initiated, the expression of cellular E-cadherin substantially decreases, while the expression of protein markers representing the morphology of mesenchyme, such as N-cadherin and Vimentin, substantially increases, and with the changes in the levels of these proteins, the mobility of the cells and the degradability of the basement membrane consequently increase, ultimately facilitating the spread of the cancer cells to the surrounding stroma [5].

The molecular mechanisms of EMT regulation in gastrointestinal malignancies are complex and regulated by multiple molecules and regulatory networks. In addition to the typical regulation of EMT, epigenetic modification, and post-translational regulation, non-coding RNAs, such as microRNAs (miRNAs) and long non-coding RNAs (lncRNAs), are important regulatory molecules. Additionally, circular RNAs (circRNAs) are important members of the non-coding RNA family and affect the invasion and metastasis of gastrointestinal malignancies by regulating the EMT of cells [6]. In this review, we described the regulatory role and molecular mechanism of circRNAs on EMT in gastrointestinal malignancies from the aspects of EMT-inducing transcription factors (TFs), EMT-related signaling pathways, and tumor microenvironments (TMEs) for a better understanding of the metastasis mechanism in gastrointestinal malignancies, as well as new perspectives for prevention and treatment strategies.

### Biological roles of circular RNAs (circRNAs) in GI malignancies

CircRNAs are a class of closed-loop RNA molecules formed by alternative splicing of precursor mRNAs and covalently linked during the 5′ and 3′ ends, which are widely found in eukaryotic cells [7]. Unlike most eukaryotic mature mRNA molecules, circRNAs do not have the typical 5′-terminal m7GTP cap structure and 3′-terminal poly(A) tail, are not easily degraded by ribonuclease (RNase), and have good stability [6]. In addition, circRNAs are relatively tissue, sequence, and disease specific and are closely associated with cancers.

**Figure 1:**
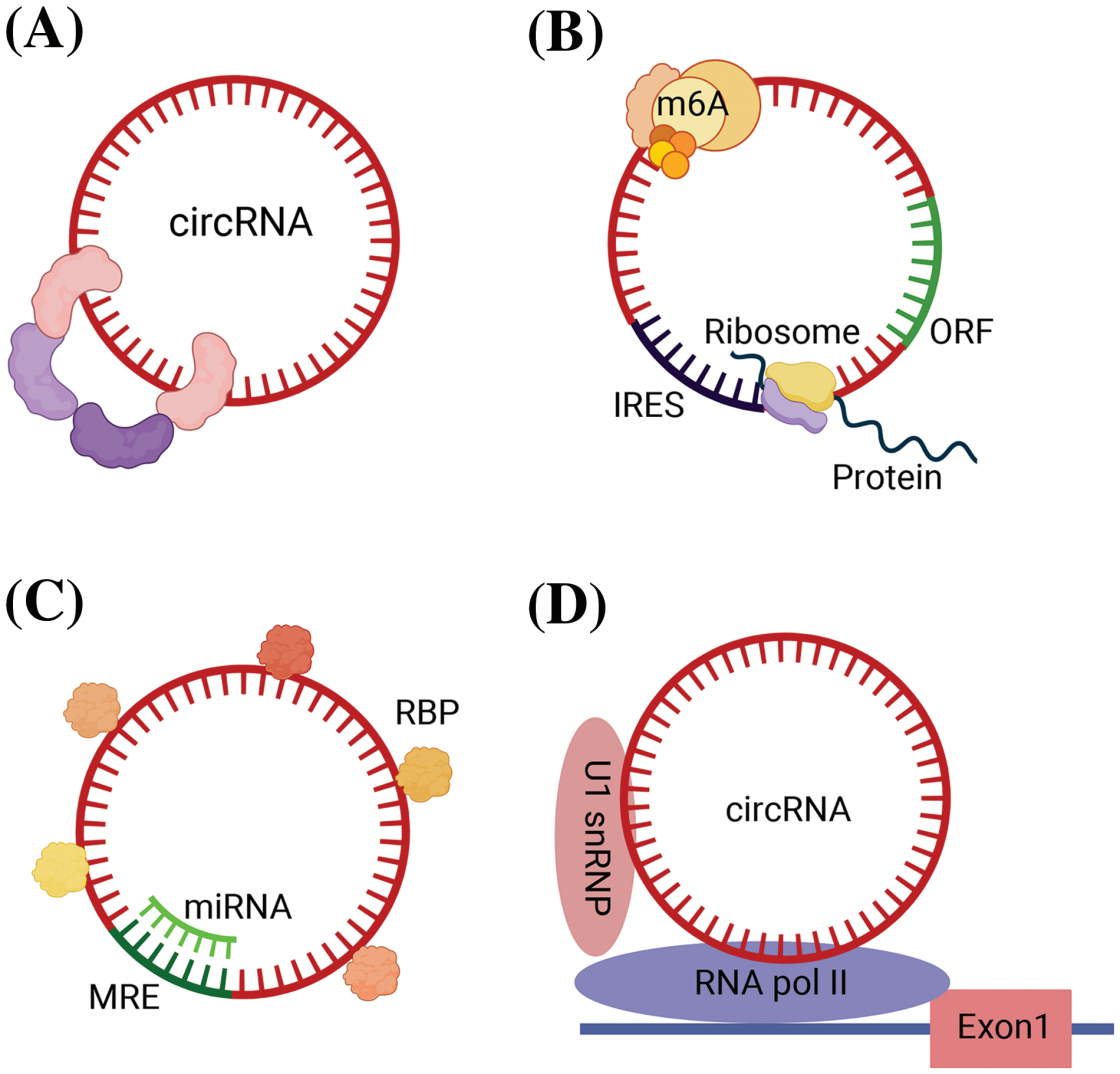
Biological roles of circRNAs in GI malignancies. (A) Function as protein scaffolds to facilitate or inhibit the assembly of protein complexes. (B) Translating into proteins due to IRES and ORF sequence and methylation modification of circRNAs. (C) Function as miRNA sponge and combining with RBP. (D) Regulating transcription. circRNA, circular RNA; GI, gastrointestinal; miRNA, micro RNA; RBP, RNA-binding proteins; IRES, internal ribosome entry site; ORF, open reading frame; MRE, miRNA response elements; m6A, N6-methyladenosine; Exon, exoniensis; U1 snRNP, U1 small nuclear ribonucleoprotein.

In the field of gastrointestinal malignancies, circRNAs are extensively involved in the regulation of gene expression at the epigenetic, transcriptional, and post-transcriptional levels through interactions with RNAs or proteins, which affects tumorigenesis and development, and is closely related to cancer invasion and metastasis, as well as to the prognosis of patients, and plays an important role in the evolution of gastrointestinal malignancies [7,8]. More and more molecular functions of circRNAs have been explored:

(1) Acting as protein scaffolds to facilitate or inhibit the assembly of protein complexes (Fig. 1A) [9]. For example, circ-Foxo3 regulate the formation of ternary complexes with proteins cyclin-dependent kinase 2 (CDK2) and cyclin-dependent kinase inhibitor 1 (or p21), thereby repressing cell cycle progression [10].

(2) Serving as templates for translation, producing functional peptides or proteins in certain contexts. CircRNAs are enriched in methylation-modified adenines, and this methylation modification play a similar role to that of ribosomal entry site sequences, which enables circRNAs to have the ability to be translated into polypeptides and proteins, and play corresponding tumor suppressor or oncogenic roles [11]. Several circRNAs are also capable of directly guiding protein translation due to the presence of internal ribosome entry site (IRES) and open reading frame (ORF) sequences (Fig. 1B). For example, circ-E-Cad encodes a protein named C-E-Cad that promotes the proliferation and migration of gastric cancer via the TGF-β/Smad/C-E-Cad/PI3K/AKT pathway [12,13].

(3) Sponging or binding RNA-binding proteins. CircRNAs are structurally equipped with miRNA response elements (MREs) and can act as miRNA sponges to regulate the expression of downstream target genes by adsorbing specific miRNAs and preventing the binding of miRNAs to downstream mRNA targets in a base-complementary pairing manner (Fig. 1C) [14]. For example, CDR1as contains more than 70 miR-7 binding sites, which can adsorb a large amount of miR-7 by exerting the function of miR-7 sponge, reducing the negative regulation of miR-7, thus indirectly regulating the expression of miRNA downstream target genes to inhibit tumor progression [15].

In addition to MRE, some circRNAs also have one or more RNA binding protein (RBP) binding sites, which can act as protein sponges to bind proteins directly or associate with proteins indirectly under the mediation of RNAs. The RNA-protein complex regulates the interaction between RNA and RBP and participates in variable splicing of RNAs, affecting protein functions and post-transcriptional gene expression (Fig. 1C). For example, circ_0088300 was proved to upregulate the RNA binding protein BOLL, promoting gastric cancer growth and EMT [16].

(4) CircRNAs modulate transcription or epigenetic activities. Some circRNAs can localize to the nucleus, which can bind to Pol II, and binds to U1 small nuclear ribonucleoproteins (snRNPs), regulating the transcriptional activity of the host (Fig. 1D) [17]. For example, the 113-aa protein (p113) encoded by hsa_circ_30402 interacts with Zuotin-related factor 1 (ZRF1) and bromodomain protein 4 (BRD4) to form a transcriptional regulatory complex inducing lipid metabolic repro-gramming and mitochondrial complex I activity, which enhances the oncogenic effects of neuroblastoma cells [18].

## Molecular Mechanism of circRNAs in EMT of GI Malignancies

### CircRNAs involvement in EMT regulation through EMT-TFs

**Figure 2:**
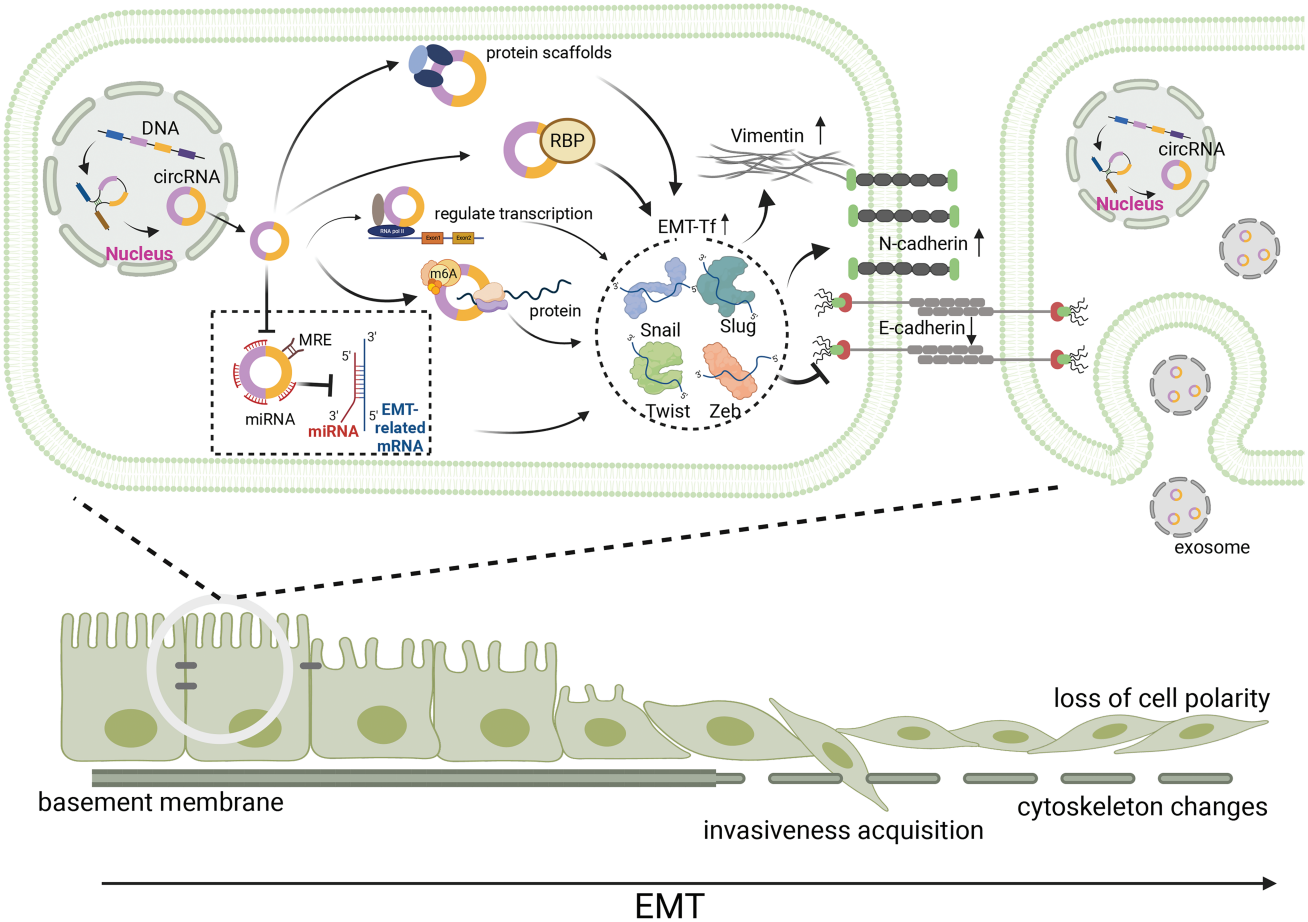
During EMT, circRNAs affect EMT-induced transcription factors, including Snail, Slug, Twist, and Zeb in a variety of ways, and further affected EMT-related molecules, such as E-cadherin, N-cadherin and Vimentin. Hence, epithelial cancer cells with reduced adhesion to each other loss cell polarity, acquire invasivness and become mesenchymal cell. Meanwhile, the basement membrane disrupted and the adhesion between the basement membrane and cells reduced, leading to cytoskeleton changes. circRNA, circular RNA; EMT, epithelial-mesenchymal transition; miRNA, micro RNA; RBP, RNA-binding proteins; m6A, N6-methyladenosine.

Epithelial to mesenchymal transition-inducing EMT-TFs are important components of EMT, and a variety of circRNAs can participate in the regulation of EMT in gastrointestinal malignancies by activating EMT-TFs [2]. These EMT-TFs include Snail (Snai1), Slug (Snail2), Twist, and Zeb, all of which can inhibit E-cadherin expression by recognizing E-box sequences, leading to cell adhesion detachment, loss of epithelial cell polarity, and conversion to a mesenchymal phenotype. Although different EMT-TFs widely induce the classical EMT process, they can respond to microenvironmental stimulation in a specific manner and act as molecular switches for EMT (Fig. 2) [19].

#### CircRNAs regulate snail in EMT

Snail (Snail1) is an EMT-inducing TF with a zinc-finger structure that can bind to the E-box sequence in the proximal promoter region of the E-cadherin gene and represses gene expression. Multiple signaling pathways can participate in EMT initiation and progression by synergistically activating Snail [20]. CircMET (circRNA_0082002), which is abnormally highly expressed in hepatocellular carcinoma tissues, can promote EMT progression by targeting downstream Snail overexpression by binding to miR-30-5p [21]. Snail can induce hepatic cell carcinogenesis through the circMET/miR-30-5p/Snail/DPP4 axis and serve as a TF for DPP4 to induce local immune suppression and participate in the occurrence and development of HCC [21]. In contrast, circFNDC3B was found to be mainly localized in the cytoplasm of intestinal cells, with reduced expression in colon cancer cells and tissues, negatively correlated with colon cancer EMT [22]. Pan et al. [22] demonstrated that circFNDC38 inhibited the process of EMT by encoding a novel protein, circFNDC3B-218aa, which regulates the expression of the Snail and ultimately inhibits cancer cell invasion, migration, and metastasis by decreasing the expression level of mesenchymal markers. Additionally, the low expression of circ-transportin3 (TNPO3) in GC tissues is closely related to the degree of differentiation of GC, weakening its role in stabilizing MYC mRNA by acting as a protein bait for insulin-like growth factor 2-binding protein 3 (IGF2BP3), which finally inhibits the proliferation and metastasis of GC by decreasing the expression of Snail through the MYC-SNAIL axis [23]. However, hsa_circ_0023642, highly expressed in GC tissues, not only acts as a molecular sponge for miR-223-3p, but also regulates the expression of Snail-associated E-cadherin, N-cadherin and Vimentin involved in the process of EMT, promoting cell proliferation, invasion, and migration, and showing a highly positive correlation with the malignant progression of GC [24,25]. Notably, another study found that miR-223-3p targeted SORBS1 to regulate the levels of E-cadherin, N-cadherin, and Vimentin to accelerate the progression of EMT in GC [26].

#### CircRNAs regulate slug in EMT

The zinc finger TF, Slug (also known as Snail2), is another member of the Snail superfamily, which is highly homologous to Snail [27]. Unlike Snail1, which exists as a monomer, Slug is mostly polymeric in conformation, and both can bind specific DNA sequences according to their relative concentrations and cellular environments to inhibit the expression of E-cadherin, which plays an important role in the process of cancer EMT [28]. Slug exogenous overexpression induces effects independent of endogenous Snail expression, which can act as an independent TF to regulate EMT induction [28]. CircRNA-0008717, whose expression was considerably upregulated in ESCC, was positively correlated with EMT and promoted the increase of Slug expression through miR-203 sponging, which ultimately promotes ESCC cell proliferation, migration, and invasion by affecting the levels of E-cadherin and Vimentin [29]. Wong et al. [30] demonstrated that the overexpression of circRTN4 in pancreatic ductal adenocarcinoma regulates miR-497-5p to promote oncogenic lncRNA HOTTIP expression, blocks ubiquitination of EMT driver RAB11FIP1, inhibits cellular RAB11FIP1 degradation, and ultimately promotes EMT, cancer growth, and liver metastasis by altering the levels of TFs, such as Slug. Additionally, bioinformatics analysis revealed that the abnormal high expression of hsa_circ_0001020 in GC could be involved in GC development through the potential p53 signaling pathway [31]. Notably, p53, a key molecule tumor suppressor in the p53 signaling pathway, was in turn confirmed to interact with MDM2 to form the p53-MDM2-Slug complex, which promotes MDM2-mediated Slug degradation and ultimately inhibits cancer invasion and metastasis [32]. The above study suggests a potential novel mechanism by which hsa_circ_0001020 regulates Slug via the p53 signaling pathway to participate in EMT, invasion and metastasis of GC.

#### CircRNAs regulate twist in EMT

Twist1 and Twist2, which belong to the basic helix-loop-helix (bHLH) TF family, are important EMT-TFs [33]. Unlike other bHLH TFs, Twist1 and Twist2 can regulate E-cadherin and Vimentin expression by binding to the E-box enhancer, which directly or indirectly affects the EMT process [34]. Meng et al. [35] found that Twist1 could directly bind to the Cul2 promoter and selectively promote the expression of Cul2 circRNA (circ-10720) in metastatic hepatocellular carcinoma, which was closely associated with tumor malignance and poor prognosis. Further luciferase assay revealed that miR-490-5p is the main adsorbing mRNA mediating circ_10720 upregulation, which promotes the expression of Vimentin and thus induces EMT [35]. Chen et al. [36] confirmed that circ_67835 was highly expressed in hepatocellular carcinoma cell lines and tissues, which could be used as a prognostic indicator of overall survival in patients, was also found to be the miR-1236-3p sponge to alleviate the inhibitory effect of miR-1236-3p on Twist2. Silencing circ_67835 promotes E-cadherin expression to inhibit the occurrence of EMT, suggesting that circ_67835 could be involved in the EMT of HCC through the miR-1236-3p/Twist2 axis to promote cancer cell proliferation and metastasis, which provides a new explanation for circRNAs-mediated EMT regulation through Twist related markers [36].

#### CircRNAs regulate Zeb in EMT

The Zeb family comprises Zeb1 and Zeb2 (also known as Smad-interacting protein 1, SIP1), which have partially overlapped genes, both binding to the E-box sequence [37]. Similar to Snail and Twist, Zeb is one of the TFs downstream of the transforming growth factor β (TGF-β) signaling pathway, and TGF-β is associated with EMT progression by inhibiting the expression of the epithelial marker E-cadherin, which in turn regulates the mRNA levels of Zeb1 and Zeb2 [38]. Zeb2 is substantially associated with the progression, malignancy, and prognosis of gastrointestinal malignancies [39,40], and Zeb2 can affect the EMT process of cancers through the Wnt/β-Catenin pathway [41]. CircUBAP2 was upregulated in pancreatic cancer tissues, which enhances the expression of the key downstream gene, Zeb1, through the inhibition of hsa-miR-494 [42]. Meanwhile, miR-494 can target SDC1 to mediate both mRNA and protein expression of E-cadherin and Vimentin, suppressing EMT, metastasis and invasiveness of pancreatic adenocarcinoma cells [43]. Regulatory T cells (Tregs) can inhibit other immune cells from generating anti-tumor immune responses, which can promote cancer cell growth when they undergo activation [44]. Zhao et al. [42] found that Treg markers, such as FOXP3, CCR8, and signal transducer and activator of transcription (STAT)5B were positively correlated with the expression level of Zeb1, suggesting that circUBAP2 can influence the EMT of pancreatic cancer cells through the regulation of miR-494 and target Zeb1 to affect the activity of Tregs for cancer progression. Zhu et al. [45] revealed that circLONP2, highly expressed in the esophagus, can upregulate the expression level of Zeb1 through miR-27b-3p sponging, as well as affect EMT-related proteins to promote the proliferation, migration, and EMT of ESCC cells. Similarly, as a circRNA abnormally highly expressed in ESCC, circ-ZDHHC5 can promote the expression of Zeb1 by binding miR-217, accelerating cell proliferation, migration and invasion [46]. MiR-217 also proved to be a molecular sponge for circRNA_100367 and can target Snail to reduce E-cadherin expression in radiation-resistant ESCC cells with high EMT ability, suggesting that circ-ZDHHC5 has the potential to regulate Zeb1 in the EMT process in ESCC cells [47]. There are limited studies regarding the influence of circRNAs on EMT in gastrointestinal cancers through Zeb, which still need to be further explored.

### CircRNAs involvement in EMT regulation through signaling pathways

In addition to promoting EMT by regulating EMT-TFs, circRNAs in gastrointestinal malignancies can also induce EMT by regulating signaling pathways, such as TGF-β, Wnt, and Notch. In different contexts, these signaling pathways can be activated by the corresponding signaling molecules, such as TGF-β, Wnt, Notch, and STAT.

#### TGF-β/Smad pathway-related circRNAs

**Figure 3:**
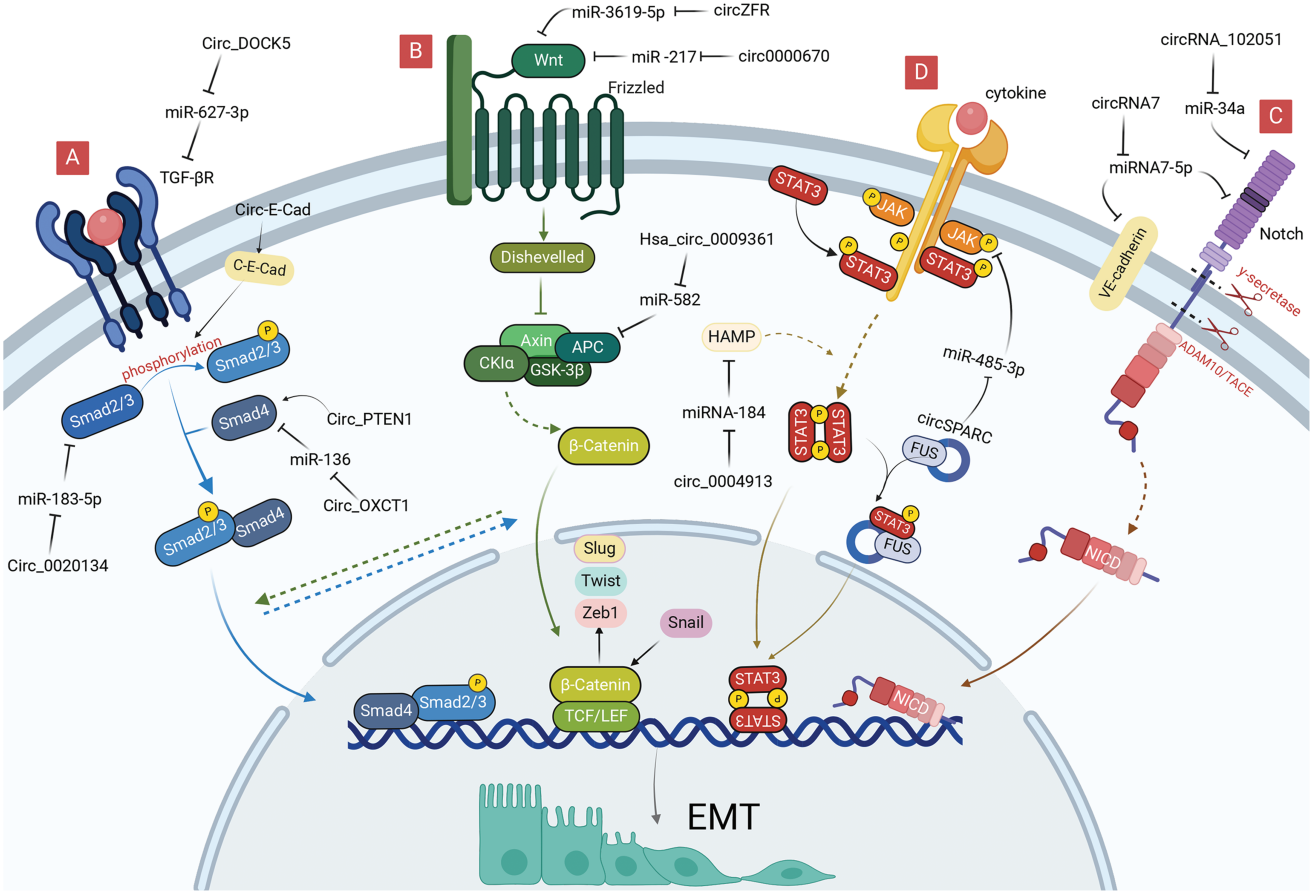
Role of circRNAs in EMT regulation via EMT-signaling pathways in GI cancer cells. (A) CircRNAs can regulate TGF-β/Smad signaling pathway to reduce EMT. (B) CircRNAs can regulate Wnt signaling pathway to reduce EMT. (C) CircRNAs can regulate Notch signaling pathway to reduce EMT. (D) CircRNAs can regulate JAK/STAT signaling pathway to reduce EMT. circRNA, circular RNA; EMT, epithelial-mesenchymal transition; GI, gastrointestinal; miRNA, microRNA.

TGF-β/Smad signaling pathway is the most classical TGF-β-mediated signaling process, considerably promoting the proliferation and metastasis of cancer cells by promoting the proliferation of mesenchymal derived cells, inhibiting the proliferation of epithelial cells and inducing EMT (Fig. 3A) [48–50]. TGFβ RI and TGFβ RII are the main receptors involved in the TGF-β/Smad pathway, which have serine/threonine protein kinase activity and regulate the transcriptional expression of downstream target genes by phosphorylating the Smad protein family. Among them, Smad4 is a key molecule in the TGF-β/Smad pathway, which can bind to phosphorylated Smad2 and Smad3 to form the Smad complex, affecting EMT-TFs and exerting a tumor-suppressive effect [51]. Inactivation, deletion or mutation of Smad4 exists in a variety of malignant tumors, and mutation of TGF-β RII has been demonstrated in gastrointestinal cancer, hepatocellular carcinoma, and gallbladder carcinoma cells, which weaken the negative regulatory mechanism of cancer cells, leading to the development of malignant cancers [52,53].

Inactivation of Smad4 promotes the deterioration and metastasis of gastric malignancies [54]. Zheng et al. [55] found that circ_PTEN1 can bind to the MH2 structural domain of Smad4 to prevent its degradation, which can allow low expression of N-cadherin and Vimentin, which suppresses EMT in colonic epithelial cells by TGF-β. Liu et al. [56] proved that downregulation of circ-OXCT1 could inhibit Smad4 expression by direct binding to miR-136, ultimately modulating the expression of E-cadherin, N-cadherin, and Vimentin through the TGF-β/Smad signaling pathway to promote cell migration, invasion, and EMT processes. CircRNA_0004872 considerably promoted downstream Smad4 gene transcript expression, which inhibited invasion and metastasis of GC cells by interacting with miR-224 [57]. Smad4 could also affect the expression level of circRNA in GC tissues through the Smad4/ADAR1/circRNA_0004872/miR-224/Smad4 feedback loop [57]. Therefore, circRNA_0004872 offers novel potential for targeting TGF-β signaling to prevent colon carcinogenesis.

The Smad2 and Smad3 are mediators of the TGF-β signaling pathway that can be mutated in a variety of cancers [58]. Little is known about the selective activation of Smad2 and Smad3. CircPVT1 serves as a sponge for miR-423-5p in GC, relieving the miR-423-5p-mediated repression of Smad3. Decreased circPVT1 inhibits GC cell EMT by increasing Smad3-related E-cadherin and downregulating Vimentin, Snai1, Twist1, and Zeb1 [59]. Additionally, circ-E-Cad could induce GC proliferation and migration by affecting the phosphorylation of Smad2 and Smad3 by encoding the circ-E-Cad protein. Moreover, circ-E-Cad overexpression upregulated Snail, Slug, and Vimentin expression, which facilitated the EMT of GC cells [13]. Moreover, silencing hsa_circ_0020134 decreases the levels of TGF-β1 and Smad2/3 protein expression in CRC, which inhibits EMT by modulating Slug, Snail, and mesenchymal markers [60].

Mutations in either the TGF-β receptor or Smad4 can abrogate signaling pathways downstream of the TGF-β receptor, similarly, can promote the cellular EMT process induced by TGF-β [61]. Meng et al. [62] discovered that circ-DOCK5, weakly expressed in ESCC, is modulated by Zeb1 and Zeb1-repressed RNA binding proteins, which can act as miR-627-3p molecular sponges, weakening the inhibitory effect of miR-627-3p on the TGFBR2, and ultimately exerting a suppressive effect on cellular EMT and metastasis. Chen et al. [63] found that circ_0087502, highly expressed in pancreatic cancer, could act as a miR-1179 molecular sponge, weakening the inhibitory effect of miR-1179 on the key target gene, TGFBR2, ultimately exerting a role in promoting cell proliferation, migration, and invasion. Yin et al. [64] discovered that circRNA_102610 could cause up-regulation of N-cadherin, Vimentin by targeting miR-103a-3p in patients with Crohn’s disease and exert EMT-promoting effects in intestinal epithelial cells. Further western blotting assays suggest that circRNA_102610 can stimulate the increase of Smad4 expression, which contradicts the inhibitory effect of Smad4 protein on EMT, requiring consideration regarding its association with an aberrant signaling pathway downstream of the Smad4 protein [64].

#### Wnt pathway-related circRNAs

The Wnt pathway is triggered by the binding of different Wnt ligands to the Frizzled family of receptors on the cell surface, including the classical Wnt, non-classical Wnt-Ca^2+^, and the Wnt-PCP pathway [65]. In contrast to the classical Wnt pathway, which needs to be mediated by β-catenin, the non-classical Wnt pathway can exert signaling via other molecules acting on the cell, such as protein kinase C and WNT5A [66,67].

The classical Wnt pathway regulates cell differentiation, proliferation, and metastasis by activating the Wnt ligand and Frizzled receptor to allow β-catenin to enter the nucleus where they function as TFs (Fig. 3B) [68]. The Wnt pathway regulates the expression of EMT-TFs by activating β-catenin to bind directly to the corresponding promoters of Slug, Zeb1, and Twist [69]. Conversely, Snail can also enhance the transcriptional activity of β-catenin, a positive feedback effect that makes Wnt signaling more readily available for cellular responses, thus promoting the use of Wnt signaling in cancer cell EMT [70].

Wnt signaling in hepatocellular carcinoma can exert an influence on c-Myc expression by regulating β-catenin, a process that is not only associated with the activation of glycolysis, but also increases the proliferation of HCC and the progression of EMT [71]. Liang et al. found that exosome circ0000670 induced by cigarettes could participate in the Wnt/β-catenin signaling pathway by positively regulating the expression levels of β-catenin and c-Myc using bioinformatics analysis [72]. Nevertheless, silencing circ0000670 resulted in a substantial decrease in the expression of EMT marker proteins and mRNAs, indicating that circ0000670 could modulate the EMT process by impacting the Wnt/β-catenin pathway [72]. Zhou et al. [73] revealed that silencing hsa_circ_0001666 in CRC inhibited cell growth and metastasis, suppressed procalcitonin 10 (PCDH10) expression by directly sponging to miR-576-5p, which reduced β-catenin expression affecting the Wnt pathway, and ultimately regulated cellular EMT by modulating key EMT proteins, such as Snail and Vimentin. Coincidentally in HCC, circUSP10 considerably upregulated and its overexpression accelerated HCC cell proliferation, migration, invasion, and EMT by sponging oncogenic miR-211-5p to regulate TCF12 expression [74]. Moreover, TCF12 is positively correlated with β-catenin in the Wnt signaling pathway, indicating that circUSP10 could regulate EMT through the Wnt pathway [75]. Tan et al. [76] discovered that downregulation of circZFR could inhibit the activity of the Wnt/β-catenin signaling pathway using the luciferase reporter and suppress the proliferation and HCC EMT by regulating the miR-3619-5p/CTNNB1 axis. Liu et al. [47] observed that radiation-resistant ESCC cells have higher EMT expression than regular ESCC cells, and silencing circRNA_100367 reduces cellular β-catenin expression and inhibits the progress of EMT. Moreover, circRNA_100367 attenuates the radio-resistance of ESCC cells via the miR-217/Wnt3 pathway. APC is a CRC anti-oncogene, and the APC protein can form a complex with β-catenin leading to the degradation of the latter when Wnt signaling is abnormal. Without the APC protein, excess β-catenin would accumulate in the nucleus, activating Wnt-targeted genes [77]. Hsa_circ_0009361 is markedly downregulated in CRC tissues, which is associated with poor prognosis, and inhibits progression, migration, invasion, and EMT of CRC cells through the miR-582/APC/β-catenin axis [78].

#### Notch pathway-related circRNAs

The Notch signaling pathway is widespread in multicellular organisms and generates active Notch fragments (Notch intracellular domain, Notch-ICD) by activating Notch ligands and receptors between neighboring cells, which enter the nucleus, binds to effector proteins and transcriptional promoters, and leads to the expression of target genes (Fig. 3C) [79]. Notch, a key oncogene or cancer suppressor gene, can regulate gene expression in a variety of ways at the transcriptional level [79], and abnormal Notch signaling is often associated with tumorigenesis [80,81]. Notch molecules were found to be significantly elevated in tumor-infiltrating regions and accompanied by the expression of EMT-TFs, such as Snail; thus the Notch signaling pathway plays an important role in regulating cancer EMT.

Some circRNAs regulate Notch transcription. CircAPLP2 is substantially upregulated in CRC. Bioinformatics analysis and luciferase reporter assays have confirmed that miR-101-3p directly interacts with circAPLP2 and targets Notch1 [82]. MiR-101-3p can also reduce the resistance of colon cancer cells to radiation and EMT progression by regulating the expression of E-cadherin and Vimentin, which provided new possibilities for circRNAs to affect EMT through the Notch pathway and a new therapeutic target for colorectal malignant tumors [83]. Notably, bromodomain PHD-finger TF (BPTF) is an attractive target for certain cancers, considerably correlating with the expression levels of EMT markers (Vimentin and E-cadherin), which can promote EMT progression in CRC [84]. Chen et al. [85] illustrated that the substantially upregulated hsa_circRNA_102051 is involved in the tumorigenesis of CRC by activating as a miR-203a sponge, which alleviates the inhibitory effect of miR-203a on Notch1 and BPTF and enhances the stemness of the cancer cells, indicating that hsa_circRNA_102051 may regulate EMT through the miR-203a/Notch1 axis. Bao et al. [86] reported that the expression level of circRNA7 in hepatocellular carcinoma was considerably lower than that in a healthy liver. Vascular endothelial cadherin (VE-cadherin) is one of the key molecules maintaining the adhesion of vascular endothelial cells, promoting EMT and cancer metastasis [87]. Notably, circRNA7 can negatively regulate the expression of VE-cadherin and Notch4 by directly targeting their 3′-untranslated region (UTR) via miRNA7-5p, which ultimately enhances EMT and promotes HCC metastasis [86]. Notably, circRNAs are associated with hepatitis B virus (HBV) infection [88]; circ_00059686 can act as a miR-129-5p sponge to inhibit the progression of HBV-associated HCC through the Notch1 pathway [89]. Currently, there is limited research related to circRNAs regulation of EMT in gastrointestinal malignancies through the Notch pathway, requiring further experimental investigation.

#### JAK/STAT pathway-related circRNAs

JAK kinases are a class of non-receptor tyrosine kinases that can be activated by cytokine signals and regulate a variety of biological activities *in vivo* using STAT as a substrate [90]. Its downstream STAT3 is a key signal involved in promoting the survival of cancer cells, and activated STAT3 enters the nucleus to enhance the transcription of downstream EMT-related genes, thereby enhancing the migration, invasion, and chemical resistance of cancer cells (Fig. 3D) [91].

To date, only a few circRNAs exert oncogenic or oncostatic effects through the JAK/STAT3/EMT axis. HAMP, a gene encoding ferredoxin, can exert cancer suppressor effects in the proliferation and migration of hepatocellular carcinoma cells through the STAT3 pathway [92]. Wu et al. [93] demonstrated a substantial decrease in circ_0004913 and HAMP in HCC and identified their target correlation with miRNA-184. Furthermore, circ_0004913 not only inhibits the JAK2/STAT3 signaling pathway through the miR-184/HAMP axis, but also suppresses EMT by reducing the expression of Snail and Vimentin [93]. CircSPARC expression is markedly upregulated in CRC tissues and promotes cell proliferation and migration, which promotes the expression of the JAK2 mRNA by sponging miR-485-3p. This circRNA could bind to the RNA-binding protein, FUS, to stimulate the translocation of phosphorylated STAT3 (p-STAT3) with RNA-binding proteins of the cell nucleus [94]. There is a positive correlation between p-STAT3 and EMT marker expression in CRC [95]. Moreover, miR-485-3p promotes EMT by enhancing the expression of Vimentin, N-cadherin, and Snail [96]. CircSPARC could therefore act as a ceRNA affecting the JAK2/STAT3 pathway to regulate the cellular EMT progression. The abnormally high expression of hsa_circ_0000117 in GC tissues and cells was negatively correlated with miR-337-3p and regulated the downstream target gene, STAT3, to enhance GC cell carcinogenesis [97]. Further bioinformatics analysis revealed that miR-337-3p could act as a molecular sponge to influence the proliferation and EMT of GC [98].

### CircRNAs involvement in EMT regulation in the TME

The TME refers to the internal and external environment where cancer cells survive, and oncogenic alterations are induced systemically and locally through endocrine and paracrine secretion [99]. In addition to malignant tumor cells, the TME contains numerous cells, including vascular endothelial, stromal, intrinsic immune (tumor-associated macrophages (TAMs) and natural killer (NK) cells), and acquired immune cells (T and B lymphocytes), whose metabolites, with cytokines included, not only serve as a source of energy supply, but also mediate various cellular messaging [100,101]. The interactions between tumor cells and the TME, mediated by exosomes, directly determine the degree of tumor malignancy [102]. As a stable exosome, circRNAs are able to influence different tumorigenic pathways in the TME, such as angiogenesis and immunosuppression, which ultimately induces EMT in cancer cells [103].

#### CircRNAs inhibits T cell activation

**Figure 4:**
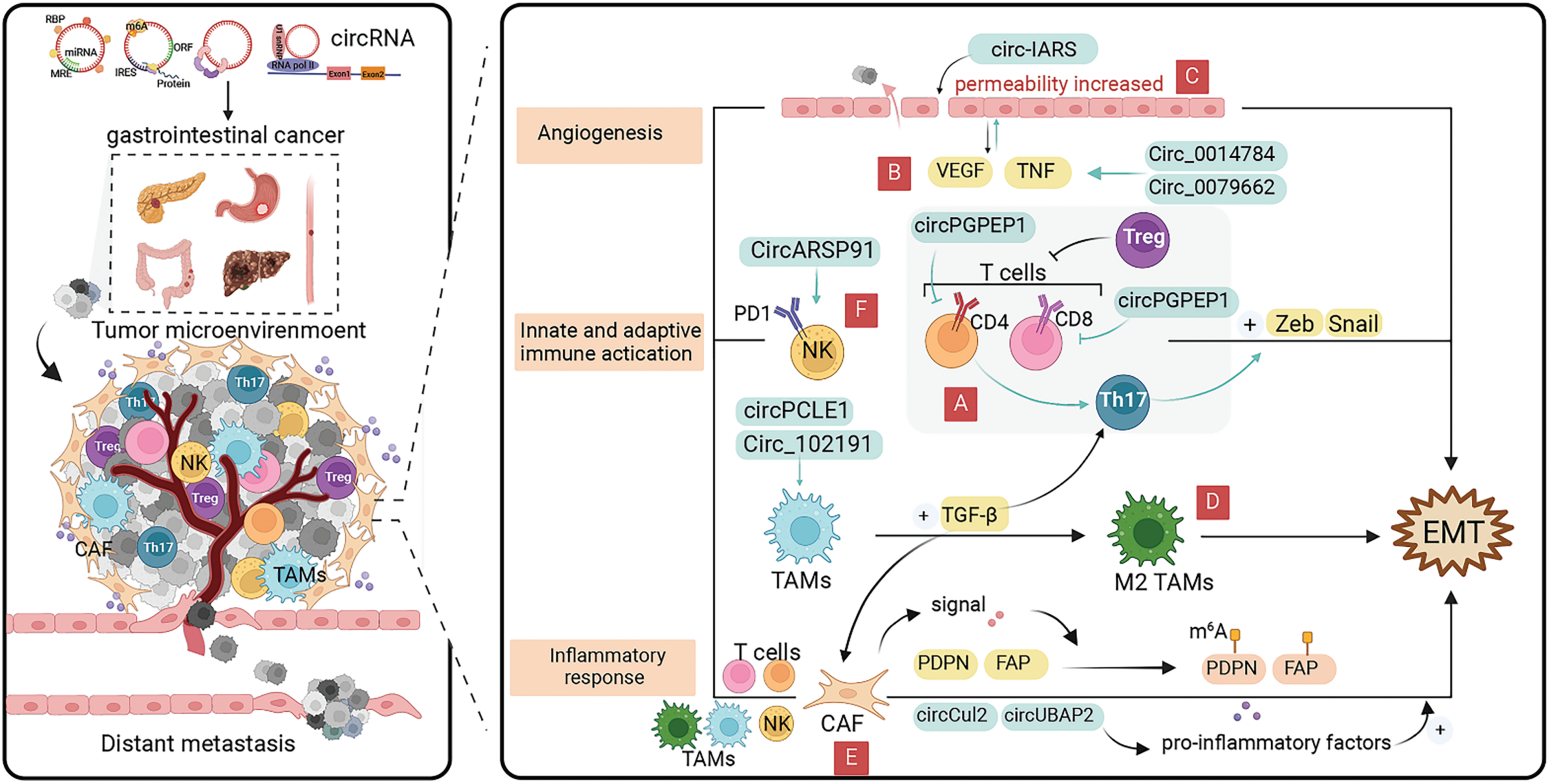
Role of circRNAs in EMT regulation via the tumor microenvironment in GI cancer cells. (A) CircRNAs can affect the EMT process by inhibiting T cell activation. (B) CircRNAs can affect cancer angiogenesis by interacting with the pro-angiogenic factors, such as vascular endothelial growth factor (VEGF) and TNF-α ereby inducing cancer EMT progression. (C) CircRNAs can promote cancer cell proliferation and EMT occurrence by regulating endothelial cell permeability. (D) CircRNAs can promote the conversion of tumor-associated macrophages to M2 phenotype for cancer progression. (E) CircRNAs can induce cancer EMT by regulating cancer-associated fibroblasts which secrete pro-inflammatory factors. (F) CircRNAs can affect the EMT process by modulating NK cell-mediated cytotoxicity. circRNA, circular RNA; EMT, epithelial-mesenchymal transition; GI, gastrointestinal; miRNA, micro RNA; NK, natural killer.

As a major component of adaptive immunity, T cells are usually involved in immune surveillance and immune editing in cancer, and the exosomal circRNAs-induced inhibitory mechanism of T cell activation can lead to immune escape and promote cancer metastasis (Fig. 4A) [104]. Zhang et al. [105] confirmed the overexpression of circPGPEP1 and NFAT5 in CRC and their target relationship with miR-515-5p. CircPGPEP1 not only increases proliferation and migration by regulating NFAT5, but also promotes the expression of Snail and N-cadherin, which are target genes of the EMT. Moreover, silencing promotes the proliferation of CD8+ and CD4+ cells and participate in the immune escape of CRC cells, indicating that circRNAs could promote EMT by promoting T-cell dysfunction and weakening the immunoreactivity of cancer cells. A similar manifestation was found in HCC, where exosome circCCAR1 expression was upregulated in hepatocellular carcinoma cells, which not only reduced the levels of perforin and granzyme B proteins in CD8+ T cells to promote their apoptosis, but also reduced the secretion of cytokines on their surface, causing dysfunction of CD8+T cells [106]. CircCCAR1 can act as a molecular sponge of miR-127-5p targeting Wilms’ tumor 1-associating protein (WTAP) molecules to promote liver cancer cell growth and metastasis [106]. MiR-127-5p overexpression in hepatocellular carcinoma cells enhances EMT progression by accelerating the activation of the SHC3/ERK signaling pathway to upregulate the expression of Vimentin and N-cadherin, suggesting that circRNAs could promote EMT by accelerating T-cell apoptosis [107]. Paradoxically, in the activated state of effector T cells, pancreatic ductal epithelial cells have substantially reduced E-cadherin expression, and markedly decreased expression of Vimentin and Zeb1, which are manifested in the form of spindle-shaped mesenchymal morphology cells, ultimately facilitating EMT [108].

#### CircRNAs-induced angiogenesis in cancers

Angiogenesis within cancers is a complex process that is co-regulated by multiple factors and plays an important role in cancer growth and metastasis [109]. Cancers can not only obtain sufficient nutrients and remove metabolic wastes through angiogenesis, but also produce a variety of vascular growth factors to further promote the expansion of the vascular network [110]. The major pro-angiogenic factors in the TME include vascular endothelial growth factor (VEGF), platelet-derived growth factor, tumor necrosis factor α (TNF-α), and interleukin (IL)-8 [111]. In the TME, exosomal circRNAs can affect cancer angiogenesis by interacting with the above vascular growth factors, thereby inducing cancer EMT progression (Fig. 4B).

EMT-induced, VEGF-A-mediated angiogenesis has been identified as a connecting mechanism between cancer stem cells and initiation [112]. Liu et al. [113] found that hsa_circ_0014784 not only promotes angiogenic differentiation of vascular endothelial cells via sponge adsorption of miR-214-3p, but also induces the target gene, YAP1, to promote pancreatic cancer cell proliferation, invasion, and EMT. Circ-BANP, which was abnormally highly expressed in hepatocellular carcinoma cells, could promote the expression level of target gene, TLR4, through sponge adsorption of miR-let-7f-5p, and further regulate the expression of VEGF-A and VEGF-2 through the STAT3 signaling pathway to affect the cancer angiogenesis [114]. Additionally, circ-BANP could weaken the inhibition of miR-let-7f-5p on Vimentin and N-cadherin to promote the occurrence of EMT, and accelerate the proliferation and migration of HCC cells [114]. HUR is a post-transcriptional regulator that can bind to a variety of mRNAs to exert different biological functions and play a promotional role in tumorigenesis and development [115]. Upregulated expression of hsa_circ_0000936 in GC tissues and serum could sponge adsorb miR-582-3p to alleviate the inhibitory effect on HUR and enhance the stability of VEGF mRNA, which ultimately leads to the rapid progression of GC *in vitro* and *in vivo* [116]. Moreover, silencing miR-582-3p could reduce Vimentin expression by promoting E-cadherin expression through the Wnt/β-catenin pathway and inhibit cancer cell EMT [117].

Exosomal circRNAs also regulate endothelial cell permeability promoting EMT in cancer cells (Fig. 4C) [118]. EMT-capable cancer cells tend to regulate endothelial cell permeability and facilitate cancer cell endocytosis during the pre-invasive phase, leading to distant cell metastasis [119,120]. Tight junction protein, Zo-1, is the site of intercellular junctions, as one of the EMT markers, its expression level is negatively correlated with the occurrence of EMT [120]. Li et al. [121] found that circ-IARS, which was substantially upregulated in plasma exosomes from pancreatic cancer tissues and patients with metastatic cancer, not only enhances endothelial single-molecule cell permeability and promotes cancer cell invasion and metastasis, but also directly binds to miR-122, promoting pancreatic cancer cell EMT by inhibiting downstream ZO-1 expression. The carcinomatous bile-derived exosome circ_CCAC1 can promote cancer progression through sponge adsorption of miR-514a-5p and be translocated to endothelial monolayers, disrupting the integrity of the endothelial barrier and inducing angiogenesis [122]. Silencing miR-514a-5p can reduce E-cadherin expression, promote Vimentin and N-cadherin expression, and induce the EMT process in cancer cells. Therefore, circ_CCAC1 can regulate the EMT occurrence in cancer cells by affecting endothelial cell permeability [123]. A growing number of researchers have focused on the role of circRNAs in angiogenesis and EMT, and in-depth examination of the specific cellular and molecular mechanisms of exosomal circRNAs is expected to lead to the development of new therapies targeting exosomal circRNAs for anti-tumor EMT in the future.

#### CircRNAs regulates macrophage polarization

TAMs are derived from bone marrow monocytes which are the most prevalent immune cells in the TME [124]. Mediated by exosomal circRNAs, TAMs can lose their killing ability and switch from M1 to M2 phenotype [125]. M2 phenotype TAMs can induce EMT development by interactions with multiple cell types and secretion of TGF-β, thus promoting cancer progression (Fig. 4D). Yi et al. [126] found that circPCLE1 can affect EMT by promoting the upregulation of M1 macrophage markers (TNF-α and IL-6), and decreasing the expression of M2 macrophage markers (IL-10 and MRC1) to induce TAM polarization [126]. Yao et al. [127] demonstrated that tumor-derived circRNA_102191 is a competing endogenous RNA of XPR1 that absorbs miR-493-3p, ultimately promoting the proliferation, migration, and invasion of GC cells by promoting the polarization of M2-type macrophages, as well as enhanced EMT by boosting the level of Vimentin and suppressing the level of E-cadherin. Fatty acid synthase (FASN) is a multifunctional peptidase that is highly expressed in many cancers, supporting cancer cell growth and proliferation and correlating with aggressive capacity [128]. In pancreatic cancer, high expression of circ_0018909 can promote EMT by inducing polarization of M2-type macrophages and regulating miR-545-3p to promote FASN expression, which affects cancer growth, metastasis, and apoptosis [129]. Additionally, circ_0018909 also can induce the polarization of dormant macrophages (M0-type) into M2-type macrophages, ultimately affecting EMT by regulating E-cadherin, N-cadherin, and Vimentin [129].

#### CircRNAs regulates cancer-associated fibroblasts (CAFs)

CAFs are one of the major components of the TME, which not only remodel the extracellular matrix, but also promote cancer metastasis through various paracrine signaling pathways (Fig. 4E) [130]. Interleukin-6 (IL6) and interleukin-11 (IL-11) belong to the IL6 family of pro-inflammatory factors which can mediate inflammation-driven tumorigenesis. CAFs secretes IL6 and IL11 in response to a variety of stimuli including IL-1β and TGF-β, inducing EMT in tumor cells [131–134]. CircCul2 is specifically expressed in CAFs and can contribute to the production of IL-6 by regulating miR-203a-3p to activate the STAT3 signaling pathway to promote proliferation and metastasis of pancreatic cancer [135]. The upregulated miR-203a-3p has been implicated in inhibiting pancreatic cancer proliferation, EMT, and apoptosis by regulating Slug. Additionally, circCul2 could modulate the CAF-induced EMT process [136]. Cui et al. found that hsa_circ_0006646 and hsa_circ_0061395 could alleviate miRNA-targeted inhibition of IL-11 by competitive binding of miRNAs and promote EMT process in esophageal cancer [137].

CXCL11 is a member of the chemotactic cytokine superfamily that normally recruits selective T cells to mediate inflammatory responses [134]. In hepatocellular carcinoma, the level of CXCL11 secreted by CAFs was considerably elevated compared with that of normal fibroblasts, and CXCL11 stimulated the upregulation of circUBAP2 expression, which further indirectly affected the levels of IL-17 and IL-1β by inhibiting miR-4756 expression [134]. IL-1β, which is considered as a regulator of the TGF-β/Smad pathway, induces the TGF-β-related EMT [138]. Additionally, IL-17a (often referred to as IL-17, is a highly versatile cellular pro-inflammatory cytokine) reduces E-cadherin expression and promotes Vimentin and N-cadherin expression, inducing EMT via the AKT pathway, suggesting the possibility of CAFs involvement in EMT and invasive metastasis of HCC by regulating IL-17 and IL-1β through circUBAP2 [139].

In addition to secreting cytokines, signals released by CAFs can alter the methylation of certain genes, such as E-cadherin, fibroblast activation protein (FAP), and flat foot protein (PDPN) to affect cancer growth and metastasis [140–142]. PDPN and FAP regulate EMT-related proteins in a variety of gastrointestinal cancers [140,142]. CircITGB6, markedly upregulated in metastatic cancer samples, could be induced by TGF-β, and was closely associated with poor prognosis of patients with colon cancer [143]. CircITGB6 enhance the mRNA stability of PDPN by directly binding to IGF2BP3, thereby promoting EMT progression in colon cancer cells by regulating the expression of E-cadherin, N-cadherin, and Vimentin [143].

#### CircRNAs regulates NK cell-mediated cytotoxicity

NK cells are bone marrow-derived cytotoxic lymphoid stem cells with the ability to self-regulate, rapidly recognize and kill tumor cells, as well as monitor tumor cell metastasis [144]. Exosomal circRNAs are NK cell-derived and involved in the immune response against cancers [145], and some prognostic genes considerably associated with EMT markers were also negatively correlated with NK cell infiltration (Fig. 4F) [146].

Hsa_circ_0048674 is substantially upregulated in HCC and exhibits sponge adsorption of miR-223-3p to regulate PDL1 expression for hepatoma cell growth, migration, and invasion [147]. Additionally, PDL1 can mitigate the effects caused by circRNAs through the PDL1/circRNA_0048674/miR-223-3p/PDL1 feedback pathway [147]. MiR-223-3p inhibits EMT occurrence in hepatocellular carcinoma cells by affecting E-cadherin and Vimentin expression through downregulation of the expression of target gene, FAT1 [148]. Silencing hsa_circ_0048674 also can promote NK cell-mediated cytotoxicity, indicating that hsa_circ_0048674 could participate in EMT process by accelerating NK cell exhaustion [147]. Ma et al. [149] found that circARSP91 could promote the expression of UL16-binding protein 1 (ULBP1) in hepatocellular carcinoma cells at both the mRNA and protein levels, affecting NK cell activation and reinforcing the sensitivity of hepatocellular carcinoma cells to NK cytotoxicity. As a major member of the NKG2D ligand family, ULBP1 plays an important role in NK cell-mediated immune response, which is expressed at a substantially lower level in the mesenchymal phenotype circulating cancer cells than that in the epithelial phenotype, suggesting that circARSP91 may be involved in EMT by altering cytotoxicity of NK cells on hepatocellular carcinoma cells [150]. Intercellular adhesion molecule 1 (ICAM-1) is expressed in concert with EMT-related proteins [151]. High expression of hsa_circ_0007456 in hepatocellular carcinoma cells considerably enhanced susceptibility to NK cells and modulated the expression of downstream target gene, ICAM-1, by directly combining with miR-6852-3p, closely related to EMT regulation [152].

### Other molecular mechanisms

**Figure 5:**
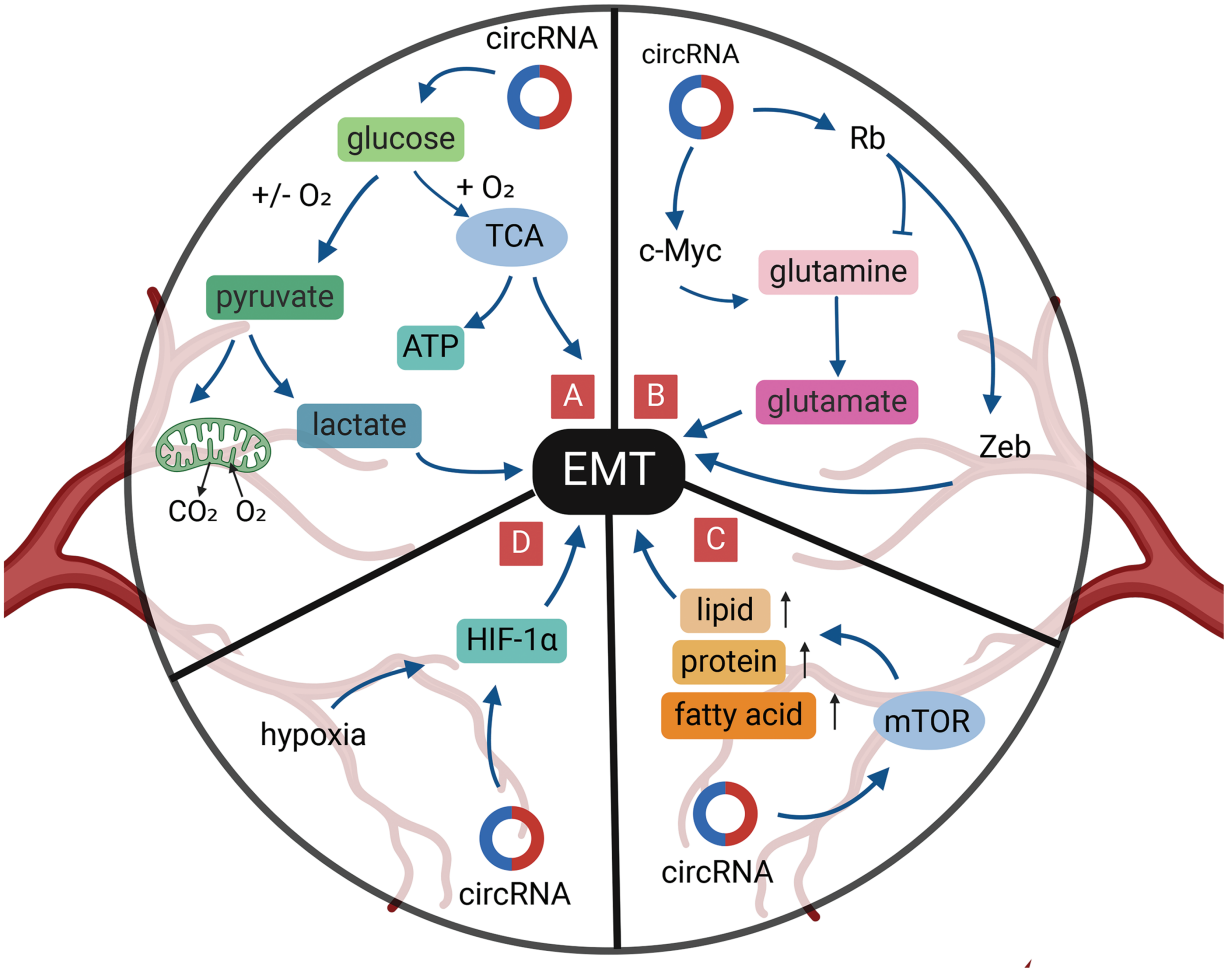
Role of circRNAs in EMT regulation via the tumor metabolism in GI cancer cells. (A) CircRNAs can regulate glucose metabolism to reduce EMT. (B) CircRNAs can regulate glutamine metabolism to reduce EMT. (C) CircRNAs can activate mTOR signal to reduce EMT. (D) CircRNAs can reduce EMT under hypoxia. circRNA, circular RNA; EMT, epithelial-mesenchymal transition; GI, gastrointestinal; miRNA, micro RNA; mTOR, mammalian target of rapamycin; TCA, tricarboxylic acid cycle; ATP, adenosine triphosphate.

Cancer is also known as a metabolic disease, where cancer cells need access to sufficient nutrients to maintain viability and biosynthesis. Among them, glucose and glutamine are two major nutrients. Cancer cells consume substantially more glucose compared with healthy cells [153,154]. Unlike healthy cells, cancer cells tend to use glycolysis as the main energy source in both aerobic and anaerobic environments, which is known as the “Warburg effect” [155]. Glycolysis can provide sufficient energy for cancer invasion, EMT, and metastasis (Fig. 5A). CircGOT1, whose expression is upregulated in esophageal squamous cell cancers, is able to promote downstream GOT1 expression through direct binding to miR-606, which ultimately affects E-cadherin, N-cadherin, and Vimentin expression for cellular EMT [156]. Additionally, silencing of circGOT1 reduces the consumption of glucose, inhibits acid-lactic acid and ATP synthesis, thus impacting on the process of glycolysis [156]. Uncoincidentally, circVPS33B disrupts oncogenic miR-873-5p in GC cells. Silencing circVPS33B inhibited the uptake of glucose and synthesis of lactic acid, which affected the Warburg effect, as well as promoted the EMT and migration of invasive GC cells by targeting HNRNPK [157].

The generation of ATP in cancer cell mitochondria involves two key metabolic pathways: the tricarboxylic acid cycle (TCA cycle) and the electron transport chain (ETC). Succinate Dehydrogenase (SDH), as the sole mitochondrial inner membrane protein in the TCA cycle, acts as a bridge between these two important metabolic pathways. SDH catalyzes the oxidation of succinate to fumarate and couples electrons to ubiquinone in the respiratory chain. Inactivation of SDH in tumors leads to the accumulation of succinate, which can promote tumor EMT [158]. It was found that circSDHAF2 translates a heterodimer of SDHAF2, an essential subunit of SDH, with a modified fifth helix, impairing the activation of SDH complex. Silencing circSDHAF2 leads to defects in the SDH complex, which inhibits tumor growth, suggesting the possibility that circSDHAF2 is involved in the tricarboxylic acid cycle to promote EMT [159].

The high demand for glutamine is also seen in cancer cells [160,161] (Fig. 5B). In ESCC cells, highly expressed circ_0001273 regulates SLC1A5 by target binding to miR-622, promoting esophageal cancer cell proliferation, migration, and EMT, and is positively correlated with glutamine consumption [162]. The TF, c-Myc, is the main driver of glutamine utilization by cancer cells, and circ_0005529 increased the expression levels of c-Myc and N-cadherin by binding to miR-527, which promoted GC cell proliferation, migration, and EMT development [163,164]. In contrast, Rb tumor suppressor protein plays a negative regulatory role in glutamine uptake [165]. Moreover, dephosphorylation of the Rb gene also exerts an inhibitory effect on the development of EMT in cancer cells through the Zeb gene [166]. CircRNA_100782, downregulated in GC, is involved in gastric carcinoma development by interacting with miR-574-3p to promote the expression of the oncogene, Rb [167]. Notably, overexpressed miR-574-3p can directly bind to the 3′-UTR of ZEB1 and impinge on the expression of E-cadherin and Vimentin, ultimately promoting EMT progression in GC cells [168]. The above study indicates the possibility that circRNA_100782 regulates glutamate-ammonia metabolism and impacts EMT in GC cells by affecting the Rb and ZEB1 gene.

Mammalian target of rapamycin (mTOR) signaling can also be activated in cancer cells and alters the expression activity of some key metabolic enzymes to regulate cancer metabolism, including ribosome biosynthesis and protein, nucleotide, fatty acid, and lipid synthesis, while mTOR signaling is involved in tumor EMT (Fig. 5C) [169,170]. CircNRIP1 acts as a molecular sponge of miR-149-5p, which can promote cellular EMT through the AKT1/mTOR pathway and affect the proliferation, migration, and invasion of GC cells [171]. Downregulation of circNRIP1 also reduces the lactate content and glucose uptake capacity of GC cells and affects the energy metabolism of cancer cells [171].

Hypoxia is one of the characteristics of the TME and is closely related to patient prognosis. Cancer tissues adapt to environmental changes by activating hypoxia inducible factor (HIF), which in turn promotes the regulation of metabolism (Fig. 5D) [172]. Deletion of HIF-1α inhibits cell proliferation, migration, and EMT, induces G0/G1 cell-cycle arrest, and promotes apoptosis [173]. Liu et al. [174] revealed that circDNMT1 can promote cell proliferation and inhibit apoptosis of GC cells by adsorbing miR-576-3p and promoting the upregulation of HIF-1α expression. Silencing of HIF-1α in GC cells inhibits the level of N-cadherin expression and ultimately hinders EMT in GC cells [175]. Thus, circDNNT1 has the potential to play a role in promoting cellular EMT through the miR-576-3p/HIF-1α axis. Furthermore, circZNF91, circRNA_100859, circDNMT1, and circPRDM4 can affect the “miRNA sponge” to regulate HIF-1α in hypoxia-induced malignant behaviors including pancreatic, colon, and hepatocellular carcinomas [176–179].

## Perspectives

Recently, the establishment of the mechanism of circRNAs action in gastrointestinal malignancies has made substantial progress. These studies indicate that circRNAs are key molecules in the process of cancer invasion and metastasis, which can affect EMT and participate in the invasion and metastasis of gastrointestinal malignancies by regulating EMT-TFs, the TME, and signaling pathways, such as Wnt, TGF-β, JAK/STAT, and Notch.

However, some prominent limitations and challenges exist. First, the mechanism of EMT is very complex, involving intracellular and extracellular signaling pathways. The study of whether there are interactions between signaling pathways is still in the early stages, and most of them draw correlation conclusions. In the future, the mechanism of different pathways in EMT needs to be further explored. Second, EMT involves numerous regulatory factors, and understanding the key inducers corresponding to different types of gastrointestinal malignancies may play a decisive role in targeted therapy. Third, although a few EMT databases exist to browse the basic features of EMT-related genes in cancers, there is a lack of EMT databases specifically for circRNAs; new databases should be created and clinical data for diseases corresponding to specific circRNAs associated with EMT should be improved. Fourth, the understanding of the role of EMT-related circRNAs in cancer is incomplete, and studies of these circRNAs affecting EMT are still at the stage of a relatively single-functional mechanism, lacking relevance. Fifth, circRNAs that can act as EMT markers have not yet been identified; newer circRNAs need to be identified and marker correlation studies need to be carried out in the future using high-throughput RNA sequencing technology. Sixth, current studies on circRNAs regulating EMT are mostly focused on common gastrointestinal malignancies, rarely in some diseases such as neuroendocrine gastroeteropancreatic (GEP) cancers [180]. The relationship between circRNAs and EMT in rare tumors of the gastrointestinal system needs more attention. Seventh, the regulation of EMT by circRNAs is primarily focused on the molecular sponge mechanism currently. Only a small number of circRNAs have been identified in gastrointestinal malignancies to participate in the EMT process through translating proteins. Future researches in the field of EMT need to be more in-depth on the side of multiple mechanisms of circRNAs such as translation, transcription, and protein scaffolding. Eighth, the current clinical translational research of circRNAs is insufficient. Most studies on the EMT mechanism of circRNAs remain at the theoretical level, and little is known about the potential clinical applications of EMT-related circRNAs in gastrointestinal malignancies [181]. In the future, we need to increase translational research and design several small molecule targeted drugs targeting circRNAs that regulate EMT by altering their levels to reverse the EMT process.

## Conclusion

In summary, circRNAs affect the development of EMT in gastrointestinal malignancies through multiple regulatory mechanisms. Thus far, circRNAs are expected to be ideal biomarkers for gene therapy and prognostic assessment of gastrointestinal malignancies. However, cancer EMT is a multifactorial and multi-stage, complex process. Therefore, the study of circRNAs associated with EMT in gastrointestinal malignancies still faces many challenges.

## Data Availability

The original data in this study can be obtained from the corresponding author upon request.
